# Tissue Circular RNA_0004018 and 0003570 as Novel Prognostic Biomarkers for Hepatitis B-Related Hepatocellular Carcinoma

**DOI:** 10.3390/genes14101963

**Published:** 2023-10-20

**Authors:** Min-Kyu Kang, Gyeonghwa Kim, Jung Gil Park, Se Young Jang, Hye Won Lee, Won Young Tak, Young Oh Kweon, Soo Young Park, Yu Rim Lee, Keun Hur

**Affiliations:** 1 Department of Internal Medicine, College of Medicine, Yeungnam University, 170 Hyunchung-ro, Nam-gu, Daegu 42415, Republic of Korea; kmggood111@naver.com (M.-K.K.); gsnrs@naver.com (J.G.P.); 2 Department of Biochemistry and Cell Biology, Cell and Matrix Research Institute, School of Medicine, Kyungpook National University, 680 Gukchaebosang-ro, Jung-gu, Daegu 41944, Republic of Korea; med.aurora1106@gmail.com; 3 Department of Internal Medicine, School of Medicine, Kyungpook National University, 130 Dongdeok-ro, Jung-gu, Daegu 41944, Republic of Korea; magnolia1103@naver.com (S.Y.J.); eworldcup@gmail.com (W.Y.T.); yokweon@knu.ac.kr (Y.O.K.); psyoung0419@gmail.com (S.Y.P.); 4 Department of Pathology, School of Medicine, Keimyung University, 1095 Dalgubeoldae-ro, Dalseo-gu, Daegu 41944, Republic of Korea; hwlee@dsmc.or.kr

**Keywords:** circular RNA, hepatocellular carcinoma, hepatitis B virus, prognosis, sarcopenia

## Abstract

The clinical significance of hsa_circ_0004018 and hsa_circ_0003570 in patients with hepatitis B virus-related hepatocellular carcinoma (HBV-HCC) is unclear. We aimed to explore the clinical significance and prognostic utility of these two circular RNAs (circRNAs) in patients with HBV-HCC. Based on 86 paired tissue samples of HCC and adjacent non-HCC, the relative expression profiles of hsa_circ_0004018 and hsa_circ_0003570 were determined using quantitative real-time polymerase chain reactions. The cut-off values were the median expression of each of the two circRNAs in 86 patients with HBV-HCC. The combination group comprised patients with high levels of the two circRNAs. Clinicopathological features, body composition profiles at the L3 level, and survival rates were investigated. The expression of hsa_circ_0004018 and hsa_circ_0003570 was downregulated in HCC tissues compared with non-HCC tissues. High expression levels of hsa_circ_0003570 (hazard ratio (HR), 0.437; *p* = 0.009) and hsa_circ_0004018 (HR, 0.435; *p* = 0.005) were inversely independent risk factors for overall and progression-free survival in patients with HBV-HCC, whereas the combination group was also an inversely independent risk factor for overall (HR, 0.399; *p* = 0.005) and progression-free survival (HR, 0.422; *p* = 0.003) in patients with HBV-HCC. The combination of hsa_circ_0003570 and hsa_circ_0004018 may be a potential prognostic biomarker for HBV-HCC.

## 1. Introduction

Hepatocellular carcinoma (HCC) has the third highest cancer-related mortality and the sixth highest cancer incidence worldwide [1]. In Korea, the trends for mortality and incidence of HCC are similar to global statistics, and the most common cause of HCC is chronic hepatitis B virus (HBV) infection, which is reported to be approximately 62.2% [2]. Most HCC patients are diagnosed at intermediate or advanced stages and have a dismal prognosis due to high recurrence, metastasis, and limited curative treatment [1,3]. In addition, owing to genomic alterations resulting from the integration of HBV DNA, HBV-related HCC (HBV-HCC) with high heterogeneity is associated with an unfavorable prognosis and increased mortality [4]. Therefore, it is essential to identify potential prognostic biomarkers for HBV-HCC, but novel and reliable biomarkers have not yet been established in clinical practice.

Circular RNAs (circRNAs) have emerged as novel prognostic biomarkers in patients with HBV-HCC [5,6]. CircRNAs are endogenous circular noncoding RNAs with covalently closed linked ends, unlike linear RNAs, which are generated by the back-splicing of exons and introns on precursor messenger RNAs (mRNAs) [7,8]. Because of the lack of a 5′ cap and 3′ polyadenylated tail, circRNAs are resistant to RNase, unlike linear RNAs [7,8,9]. Although a clear role of circRNAs has not been identified, their functions have been reported as modulators of gene expression by acting as microRNAs (miRNAs) and sponging or competing with endogenous RNA at the transcriptional and posttranscriptional levels [7,8]. Furthermore, the dysregulation of circRNAs is associated with cell growth, tumor progression, invasiveness, and metastasis by altering gene expression and signaling pathways via circRNA/miRNA/mRNA in patients with HBV-HCC [4,6,10]. In in vitro studies, the high expression of hsa_circ_0004018 and hsa_circ_0003570 has been shown to be a tumor suppressor via the circRNA/miRNA/mRNA regulatory network in patients with HBV-HCC [10,11,12,13]. However, the role and clinical significance of both hsa_circRNAs as tumor suppressors in patients with HBV-HCC based on real world clinical data remains uncertain.

In this study, we evaluated the clinical outcomes, including survival and cancer progression, of hsa_circ_0004018 and hsa_circ_0003570 in 86 patients with HBV-HCC. Furthermore, we investigated the clinical significance of the combination of these two circRNAs as potential prognostic biomarkers independent of traditional risk factors.

## 2. Materials and Methods

### 2.1. Study Design and Participants

This retrospective, single-institution study investigated the clinical significance of two circRNAs (hsa_circ_0004018 and hsa_circ_0003570) as prognostic biomarkers for patients with HBV-HCC. In our previous study, 121 patients with HCC who performed diagnostic biopsies or surgical resections were included [14]. Of these, 47 patients (11 patients with anti-hepatitis C virus (HCV) positivity, 32 with excessive alcohol consumption, 2 with coinfection of HBV and HCV, and 2 with non-alcoholic steatohepatitis) who tested negative for HBV were excluded from this study. Meanwhile, a total of 84 HBV-HCC patients, 74 patients from the previous study and 14 HBV-HCC patients with additional tissue acquisition were enrolled in this study. The end date of the clinical follow-up was March 2020.

We analyzed the clinicopathological data of 86 patients with HCC, including anthropometric parameters, Child–Turcotte–Pugh (CTP) class, laboratory findings, tumor stage, and computed tomography (CT)-based body composition parameters. The diagnosis of HCC was based on noninvasive imaging modalities, including liver-specific dynamic CT and gadoxetic acid disodium-enhanced magnetic resonance imaging, or pathologic confirmation, according to the guidelines of the European Association for the Study of the Liver [3]. The Modified Union for International Cancer Control and Barcelona Clinic Liver Cancer (BCLC) classification was used for HCC staging [2,15]. Since histological confirmation of the fibrosis stage in background tissue was not available for all patients with HBV-HCC, we defined advanced fibrosis using high cut-off values of the fibrosis-4 index (≥2.67). The fibrosis-4 index was calculated as follows: age (yr) × aspartate aminotransferase (U/L)/(platelet count [10^9^/L] × alanine aminotransferase [U/L]) [16,17]. Response Evaluation Criteria in Solid Tumors (version 1.1) were used to evaluate tumor response [18]. During the follow-up period, laboratory and dynamic imaging studies were performed every 3 months to monitor each patient’s status. The primary and secondary endpoints were overall survival (OS) and progression-free survival (PFS), respectively. The OS and PFS were defined based on the results of our previous study [14].

### 2.2. Acquisition of Tissue Samples

All tissue specimens with tumors and adjacent nontumor tissues (NT) from 86 patients with HCC were immediately stored at 4 °C overnight in an RNAlater™ Stabilization Solution (Invitrogen^TM^; Thermo Fisher Scientific, Waltham, MA, USA) and then stored at −80 °C. This research was approved by the Institutional Review Board (approval number: KNUH-2014-04-056-001) and was done in accordance with the ethical guidelines of the 1975 Declaration of Helsinki. Written informed consent was obtained from all patients prior to sampling.

### 2.3. Total RNA Extraction and Complementary DNA Synthesis

Total RNA was extracted from homogenized frozen tissues obtained from 86 patients with HCC using the QIAzol^®^ Lysis Reagent (Qiagen, Hilden, Germany), according to the manufacturer’s instructions. The quantity, quality, and purity of the extracted RNA were evaluated using a NanoPhotometer^®^ N60 (Implen, Westlake Village, CA, USA).

### 2.4. Complementary DNA Synthesis and Quantitative Real-Time Polymerase Chain Reaction

Complementary DNA (cDNA) was synthesized using a high-capacity cDNA Reverse Transcription Kit (Applied Biosystems, Foster City, CA, USA), according to the manufacturer’s instructions. Quantitative real-time polymerase chain reactions (qRT-PCR) were performed using a Power SYBR™ Green PCR Master Mix (Applied Biosystems) and the QuantStudio 6 RT-PCR System (Applied Biosystems). All qRT-PCR experiments were performed in triplicates. For the analysis of relative circRNA expression, divergent primers for each of the two hsa_circRNAs, including the gap junction of circRNA, were designed as previously described [14,19]. The protein-coding gene names of hsa_circ_0004018 and hsa_circ_0003570 were SET and MYND domains containing 4 (SMYD4) and family with sequence similarity 53 member B (FAM53B), respectively. The primers’ sequences of hsa_circ_0004018 and hsa_circ_0003570 used were as follows: hsa_circ_0004018:5′- TCA ACC TTT TGC CCC ACA CT -3′ (forward) and 5′- AAG ACA CGT CTG TGT GTT GT -3′ (reverse), and hsa_circ_0003570:5′- CAA GAT GGC ACA GCA CAC GC -3′ (forward) and 5′- ATG CTG GTG CTC GGT TGG TC -3′ (reverse). The expression analysis of relative circRNAs was normalized to that of glyceraldehyde 3-phosphate dehydrogenase, and the sequences of the primers for glyceraldehyde 3-phosphate dehydrogenase were as described in our previous study [14,19]. All primers used in this study were synthesized by Bionics Co., Ltd. (Seoul, Republic of Korea). The amplification of hsa_circ_0004018 and hsa_circ_0003570 was analyzed using a melt curve. The relative expression levels of hsa_circ_0004018 and hsa_circ_0003570 were calculated and analyzed using the 2 −^ΔΔ^Ct method.

### 2.5. Evaluation of Body Composition Variables

Based on the Picture Archiving and Communications System (Centricity, GE Healthcare, Milwaukee, WI, USA), cross-sectional images at the L3 level from an abdominal CT scan were obtained. The specific software that measured the areas of the skeletal muscle mass and adipose tissues using varying Hounsfield unit thresholds was AutoMATiCA (https://gitlab.com/Michael_Paris/AutoMATiCA (accessed on 12 September 2022.)) [20]. The indices of the body composition variables were defined as the area (cm^2^) divided by the square of the height (m^2^). Sarcopenia was classified as a skeletal muscle index <50 cm^2^/m^2^ for men and <39 cm^2^/m^2^ for women [21]. The presence of visceral adiposity was defined as a visceral/subcutaneous fat ratio of >1.33 for males and >0.93 for females [22].

### 2.6. Statistical Analysis

Numerical data were expressed as means and standard deviations (SDs) for normally distributed data, while categorical data were presented as percentages. For non-normally distributed data, medians and interquartile ranges (IQRs) were reported. We used the chi-square test, Fisher’s exact probability test, and the Mann–Whitney U test to compare the clinicopathological characteristics of the groups according to the expression of hsa_circ_0004018 and hsa_circ_0003570. To examine the contrast in the expression of the two hsa_circRNAs between tumors and adjacent NT tissues, a paired t-test was performed. The combination group was defined as having a high expression of both hsa_circ_0003570 and hsa_circ_0004018. We analyzed patient survival and compared survival between the groups using the Kaplan–Meier method and log-rank test. To identify predictors of survival, we performed logistic regression analyses based on the Cox proportional hazards model. All statistical analyses were performed using the R software (version 4.1.0; R Core Team, 2021; R Foundation for Statistical Computing, Vienna, Austria), and statistical significance was set at *p* < 0.05.

## 3. Results

### 3.1. Baseline Characteristics of the Enrolled Patients

The baseline characteristics are shown in Table 1.

Of the 86 patients with HBV-HCC, 50 (58.1%) died during the follow-up period. The median age was 57.0 years (interquartile range (IQR), 52.0–65.0 years), and 73 patients (84.9%) were men. Regarding tumor profiles, 39 (45.3%) had tumors in the T3 and T4 stages, 5 (5.8%) had tumors in the N1 stage, and 9 (10.5%) had tumors in the M1 stage. The median α-fetoprotein (AFP) levels were 137.0 ng/mL (IQR, 7.5–4814.0 ng/mL). Forty-seven (54.7%) patients were found to have advanced fibrosis. Of the 86 patients, 76 (88.4%), 9 (10.4%), and 1 (1.2%) were classified as CTP-A, CTP-B, and C, respectively. On the circRNA profiles, the expression of hsa_circ_0004018 and hsa_circ_0003570 in HCC tumor tissues was 0.0003 (IQR, 0.0001–0.0006) and 0.0006 (IQR, 0.0004–0.0012), respectively.

**Figure 1:**
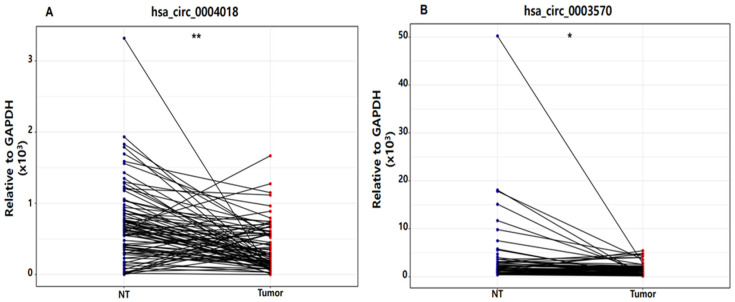
Dot plot of hsa_circ_0004018 (**A**) and hsa_circ_0003570 (**B**) expression in nontumor tissue (NT) and tumor tissue in patients with hepatitis B virus-related hepatocellular carcinoma. ** *p* < 0.001, * *p* < 0.05. GAPDH, glyceraldehyde 3-phosphate dehydrogenase.

In this study, qRT-PCR was performed on the nontumor (NT) tissues and tumor tissues of patients with HBV-HCC. The CT values of GAPDH were measured to be 17.078–19.851 in NT tissues and 17.02–19.673 in tumor tissues, respectively. We verified that there was no difference in the expression levels of GAPDH between tissue samples, and we used GAPDH as a normalizer (Supplementary Figure S1). In addition, the expression levels of hsa_circ_0004018 and hsa_circ_0003570 in HCC tumor tissues were lower than those in nontumorous adjacent tissues (hsa_circ_0004018: *p* < 0.001 and hsa_circ_0003570: *p* < 0.05: Figure 1).

Regarding body composition profiles, 48 (55.9%) and 27 (31.4%) patients had sarcopenia and visceral adiposity, respectively. The median durations of OS and PFS were 73.6 months (IQR, 12.3–133.1 months) and 34.4 months (IQR, 9.8–85.5 months).

Patients with HBV-HCC were divided into high- and low-expression groups, based on the median expression levels of the two circRNAs (Table 2). Since both hsa_circRNAs values for 86 patients did not meet the normality test, the median values in the current study were derived using the non-parametric Mann–Whitney U test. The median values of hsa_circ_0004018 and hsa_circ_0003570 were 0.0002793 and 0.0006155, respectively. The high expression group of hsa_circ_0003570 had a lower proportion of BCLC B and C stages (62.8 vs. 37.2%, *p* = 0.031) and lower AFP levels (1000 vs. 22.1 ng/mL, *p* = 0.009) than the low expression group. However, no significant differences were observed in the hsa_circ_0004018 expression group.

### 3.2. Prognostic Factors for OS in Patients with HBV-HCC, including the Two circRNAs

**Figure 2:**
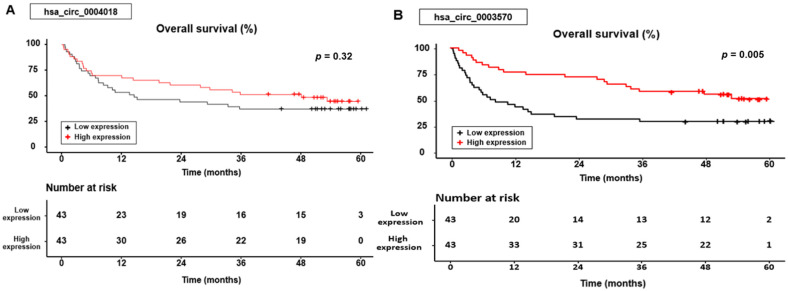
Overall survival curves for the hsa_circ_0004018 (**A**) and hsa_circ_0003570 (**B**) expression.

Based on the Kaplan–Meier curve, OS was significantly different according to the hsa_circ_0003570 expression group (*p* = 0.005), whereas the OS was not statistically significant according to the hsa_circ_0004018 expression group (*p* = 0.32) (Figure 2).

In the group expressing hsa_circ_0004018, the cumulative 1-, 3-, and 5-year OS rates were 69.8%, 51.2%, and 44.4% for patients with high expression levels, compared with 53.5%, 37.2%, and 37.2% for those with low expression levels. In the hsa_circ_0003570 expression group, the cumulative 1-, 3-, and 5-year OS rates were 76.7%, 58.1%, and 51.2% for patients with high expression levels, compared with 46.5%, 30.2%, and 30.2% for those with low expression levels.

Table 3 presents factors associated with OS in HBV-HCC. Multivariate analysis indicated that advanced T stage (hazard ratio (HR), 6.142; 95% confidence interval (CI), 3.081–12.242; *p* < 0.001), the presence of nodal involvement (HR, 3.397; 95% CI, 1.172–9.849; *p* = 0.024), metastasis (HR, 2.506; 95% CI, 1.062–5.913; *p* = 0.036), decompensated liver cirrhosis (LC) defined as CTP classes B and C (HR, 4.107; 95% CI, 1.786–9.444; *p* < 0.001), and sarcopenia (HR, 2.026; 95% CI, 1.103–3.722; *p* = 0.023) were significant risk factors for OS. In particular, high hsa_circ_0003570 expression (HR, 0.437; 95% CI, 0.235–0.813; *p* = 0.009) was found to have an inverse association with OS in patients with HBV-HCC.

### 3.3. Prognostic Factors for PFS in Patients with HBV-HCC, including the Two circRNAs

**Figure 3:**
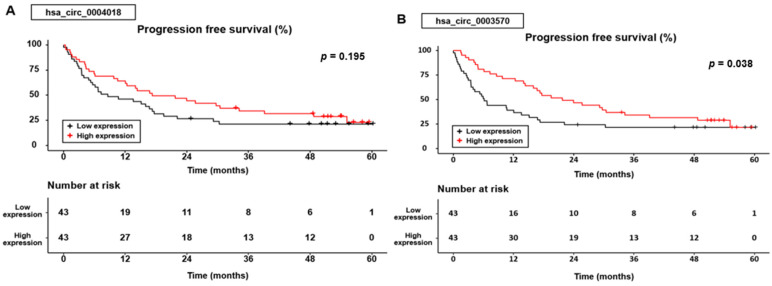
Progression-free survival curves for the hsa_circ_0004018 (**A**) and hsa_circ_0003570 (**B**) expression.

Similar to the OS results, PFS was significantly different according to the hsa_circ_0003570 expression group (*p* = 0.038), whereas the PFS was not statistically significant according to the hsa_circ_0004018 expression group (*p* = 0.195) (Figure 3). 

In the group expressing hsa _circ_0004018, the cumulative 1-, 3-, and 5-year PFS rates were 64.3%, 34.5%, and 23.2% for patients with high expression levels, compared with 46.3%, 21.4%, and 21.4% for those with low expression levels. In the hsa_circ_0003570 expression group, the cumulative 1-, 3-, and 5-year PFS rates were 71.4%, 34.3%, and 21.8% for patients with high expression levels, compared with 39%, 21.8%, and 21.8% for those with low expression levels.

Multivariate analysis revealed that advanced T stage (HR, 5.040; 95% CI, 2.547–9.974; *p* < 0.001) and decompensated LC (HR, 2.908; 95% CI, 1.345–6.285; *p* = 0.007) were associated with PFS. In addition, the presence of visceral adiposity (HR, 0.445; 95% CI, 0.238–0.830; *p* = 0.001) and the high expression of hsa_circ_0004018 (HR, 0.435; 95% CI, 0.242–0.782; *p* = 0.005) had an inverse association with PFS in HBV-HCC (Table 3).

### 3.4. Impact of Combined hsa_circ_0004018 and hsa_circ_0003570 for Predicting OS and PFS in Patients with HBV-HCC

We found that hsa_circ_0004018 was related to PFS, while hsa_circ_0003570 was related to OS in patients with HBV-HCC, independent of traditional risk factors, including tumor and liver function-related factors. To evaluate the impact of the combination of the two circRNAs, a group satisfying the high expression of both hsa_circRNAs was defined as the “combination group.” Table 4 shows the clinical values of the combination and non-combination groups. The combination group had a longer duration of PFS (59.9 vs. 20.5 months, *p* = 0.024), and tended to have a longer OS than did the non-combination group, but it was not statistically significant (119.0 vs. 33.9 months, *p* = 0.093).

**Figure 4:**
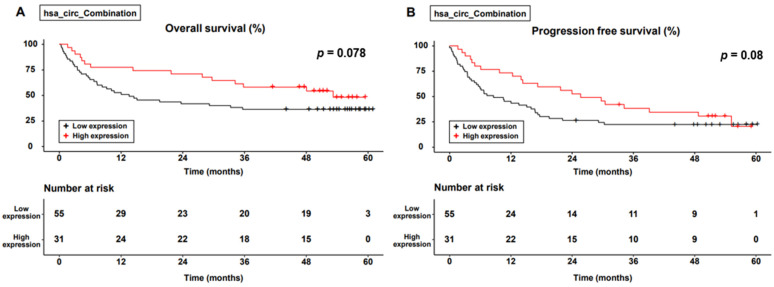
Survival curve for the level of expression of combined circular RNAs. (**A**) Overall survival; (**B**) progression-free survival.

Figure 4 shows the differences in OS and PFS between the two groups. The cumulative 1-, 3-, and 5-year OS rates were 78.1%, 56.2%, and 46.7% in the combination group and 51.9%, 37.0%, and 37.0% in the non-combination group, respectively. The cumulative 1-, 3-, and 5-year PFS rates were 74.2%, 37.0%, and 29.7% in the combination group and 44.2%, 22.8%, and 22.8% in the non-combination group, respectively. Although the combination group tended to achieve better OS and PFS than did the non-combination group, the difference was not significant (*p* = 0.078 in OS and *p* = 0.08 in PFS, respectively). 

Advanced T stage (HR, 7.613; 95% CI, 3.792–15.284; *p* < 0.001), the presence of nodal involvement (HR, 3.398; 95% CI, 1.178–9.806; *p* = 0.024), metastasis (HR, 2.645; 95% CI, 1.120–6.249; *p* = 0.027), decompensated LC (HR, 3.827; 95% CI, 1.722–8.504; *p* = 0.001), and sarcopenia (HR, 2.034; 95% CI, 1.110–3.728; *p* = 0.022) were significant risk factors for OS based on multivariate analysis. Similar to previous results, the combination group (HR, 0.399; 95% CI, 0.209–0.765; *p* = 0.005) had an inverse association with OS in patients with HBV-HCC. In addition, advanced T stage (HR, 5.253; 95% CI, 2.612–10.564; *p* < 0.001), decompensated LC (HR, 3.475; 95% CI, 1.616–7.470; *p* = 0.001), and sarcopenia (HR, 1.895; 95% CI, 1.094–3.282; *p* = 0.023) were significant risk factors for PFS. Visceral adiposity (HR, 0.435; 95% CI, 0.233–0.814; *p* = 0.009) and the high expression levels of both circRNAs (the combination group) (HR, 0.422; 95% CI, 0.237–0.751; *p* = 0.003) were found to have an inverse association with PFS in patients with HBV-HCC (Table 5).

## 4. Discussion

Compared with the expression of hsa_circ_0004018 and hsa_circ_0003570 in normal tissues, this study revealed that the expression of the two circRNAs was low in HBV-HCC tissues. We have shown that tissue hsa_circ_0004018 and hsa_circ_0003570 are associated with a favorable survival outcome including OS and PFS in patients with HBV-HCC. In addition, the combination of the two circRNAs may be a potential predictor for survival and progression in HBV-HCC patients, independent of traditional risk factors, including tumor, liver function-related, and body composition variables based on CT.

In previous studies, hsa_circ_0004018 was well known be a potential tumor suppressor in patients with HCC, which was transcribed from SMTD4 [12]. Fu et al. [12] showed that the expression of hsa_circ_0004018 was found to be lower in HCC tissues compared to adjacent nontumorous tissues. In this study, patients with HCC overexpressing hsa_circ_0004018 had favorable clinicopathological characteristics, including BCLC and TNM staging, differentiation, and AFP levels [12]. Wang et al. [23] revealed that hsa_circ_0004018 may be a novel biomarker for HCC tumor suppression by regulating miR-1197 in the PTEN/PI3K/AKT pathway. Zhu et al. [10] revealed another molecular pathway of the hsa_circ_0004018/miR-626/DKK3 regulatory axis that inhibits the Wnt/β-catenin signaling pathway, which may be a potential therapeutic target in HCC. Furthermore, hsa_circ_0004018 is regarded as a suppressor of liver fibrosis progression and angiogenesis in patients with HCC. Li et al. [24] showed that the upregulation of hsa_circ_0004018 was associated with the suppression of liver fibrogenesis by regulating the hsa-miR-660-3p/TEP1 axis, leading to a blocking of the transition from fibrosis to HCC. Wu et al. [13] demonstrated that the overexpression of hsa_circ_0004018 might serve as an angiogenesis inhibitor by binding to fused proteins in sarcomas and regulating the expression of the tissue inhibitor of metalloproteinase 2.

However, no study has identified the clinical impact of hsa_circ_0004018 in patients with HBV-HCC. Our study focused on clinical outcomes, including survival and progression, in accordance with hsa_circ_0004018 expression in patients with HBV-HCC. Similar to the results of previous studies, the expression of hsa_circ_0004018 was decreased in HBV-HCC tissues compared to that in nontumorous tissues in the current study [12,23]. In addition, we demonstrated that the high expression of hsa_circ_0004018 was inversely associated with PFS, independent of tumor, liver function-related, and body composition factors, but not with OS. This suggests that tissue hsa_circ_0004018 may be a potential prognostic marker of tumor suppressor properties for HBV-HCC.

The role of hsa_circ_0003570 in patients with HCC is relatively unknown compared with that of hsa_circ_0004018. Fu et al. [11] demonstrated that the overexpression of tissue hsa_circ_0003570 had favorable clinicopathological features, including tumor diameter, degree of differentiation, vascular invasion, staging, and AFP levels in patients with HCC. In a recent study, we found that the low expression of hsa_circ_0003570 was associated with worse outcomes, such as shorter OS and PFS. This suggests that hsa_circ_0003570 may be a potential novel prognostic biomarker for predicting outcomes in patients with HCC.

In the current study, hsa_circ_0003570 was inversely associated with OS but not with PFS, which was contrary to the findings of a previous study [11]. Similar to the expression of hsa_circ_0004018, the expression of hsa_circ_0003570 was downregulated in HCC tissues compared to that in nontumorous adjacent tissues. Hsa_circ_0003570 and hsa_circ_0004018 may be potential prognostic factors in patients with HBV-HCC.

In our study, we aimed to determine the clinical implications based on the combination of the two circRNAs with the aforementioned tumor suppressor roles. Although the molecular pathway was not directly identified in this study, the putative mechanism of the combination of the two circRNAs is considered to involve the Wnt/β-catenin signaling pathway. In a previous study, the high expression of miR-182-5p activated the Wnt/β-catenin signaling pathway by interfacing with β-catenin degradation, leading to unfavorable prognoses, including early recurrence in HCC patients that underwent surgery [25]. Recently, Zhang et al. demonstrated that the overexpression of circ_0003570 suppressed HCC progression by regulating the miR-182-5p/STARD 13 axis [26]. Similar to the tumor suppressor role of hsa_circ_0004018 by inhibiting the Wnt/β-catenin signaling pathway, hsa_circ_0003570 may function as an HCC tumor suppressor by sponging miR-182-5p, leading to the inactivation of Wnt/β-catenin signaling pathway.

Furthermore, owing to the discordance in the clinical impact of the two tissue circRNAs on OS and PFS, we investigated the clinical significance of survival and progression using a combination of the two circRNAs. Studies on the clinical significance of tissue circRNA combinations in patients with HCC are lacking. In our study, there were no differences in the tumor, liver function, and clinical outcome profiles, except for the duration of PFS in the combination group, defined as having a higher expression of hsa_circ_0004018 and hsa_circ_0003570 than in the noncombination group. However, considering that the high expression combination group was independently correlated with survival and progression in patients with HBV-HCC, the combination of the two differentially expressed circRNAs could be a method for discovering prognostic targets for HCC in the future.

Recent studies have shown that sarcopenia and visceral adiposity are related to worse survival outcomes in patients with HCC [22,27]. Similar to previous studies, we found that sarcopenia was independently associated with OS and PFS, which may be prognostic factors for HBV-HCC [22,27]. The putative mechanisms of sarcopenia in patients with HCC are complex and are related to chronic inflammation, including tumor necrosis factor-α and interleukin-6, insulin resistance, and low levels of vitamin D, which lead to the progression of liver fibrosis and HCC [22,28,29,30]. In contrast to other studies, in the current study, visceral adiposity was inversely associated with PFS but not with OS. In previous studies, high visceral adiposity was associated with poor prognoses in patients with HCC due to abundant inflammatory cytokines derived from excess visceral fat and prolonged insulin resistance [22,31]. However, a recent meta-analysis revealed that there was no relationship between visceral adiposity and worse clinical outcomes in HCC, and the role of visceral adiposity in HCC is controversial [32]. In the current study, the two tissue circRNAs were not statistically significant prognostic factors for sarcopenia or visceral adiposity.

The limitations of this study were as follows: First, it was difficult to extend the application to the entire population due to the Korean cohort-based study, small tissue sample, retrospective nature of the study, and potential for selection bias. Second, we could not identify the potential circRNA/miRNA/mRNA molecular mechanisms of these two circRNAs. Considering that the progression of sarcopenia is related to the activation of the Wnt/β-catenin signaling pathway, additional molecular studies may reveal a link between sarcopenia and the two circRNAs which inhibit the Wnt/β-catenin signaling pathway [10,33]. However, the association between the two hsa_circRNAs and sarcopenia was insignificant in the current study. Third, we could not investigate the clinical impact of the two circRNAs as circulating biomarkers in serum, plasma, or exosomes. Further research is required to clarify the roles of hsa_circ_0004018 and hsa_circ_0003570 as circulating prognostic biomarkers. Fourth, due to lack of background information using transient elastography or liver biopsy, we could not reveal an accurate value of the liver fibrosis grade. Finally, due to the small number of the patients, the area under the receiver operating characteristic (AUROC) curve of both circRNAs for survival outcome was not significant (AUROC, 0.586 in hsa_circ_0004018, 0.619 in hsa_circ_0003570, respectively). Further large cohort-based studies are warranted.

## 5. Conclusions

In conclusion, the combination of the high tissue expression of hsa_circ_0004018 and hsa_circ_0003570 is strongly associated with favorable clinical outcomes and can be a novel prognostic marker in patients with HBV-HCC. Further, larger sample sizes and well-validated studies are needed to elucidate the mechanisms of hsa_circ_0004018 and hsa_circ_0003570 and their potential as prognostic factors and circulating biomarkers for HCC.

## Figures and Tables

**Table 1:** Baseline characteristics.

Parameter	Hepatitis B Virus-Related HCC (*n* = 86)
Age	57.0 [52.0–65.0]
Male, n (%)	73 (84.9)
Body mass index, kg/m^2^	23.5 [21.7–25.7]
Liver function profiles	
Aspartate aminotransferase, IU/L	51.0 [27.0–74.0]
Alanine aminotransferase, IU/L	78.5 [43.0–96.0]
Platelet count, ×10^9^/μL	151.0 [115.0–193.0]
Total bilirubin, mg/dL	0.7 [0.5–1.2]
Serum albumin, g/dL	3.9 [3.5–4.2]
Prothrombin time, INR	1.1 [1.1–1.2]
Fibrosis-4 index	3.0 [1.8–5.0]
Tumor profiles	
Tumor number, multiple	38 (44.2)
Tumor size, >5 cm	24 (27.9)
T stage, T0,1,2/3,4	47 (54.7)/39 (45.3)
N stage, N0/1	81 (94.2)/5 (5.8)
M stage, M0/1	77 (89.5)/9 (10.5)
TNM stage, I/II/III/IV	41 (47.7)/11 (12.8)/22 (25.6)/12 (14.0)
BCLC, 0, A/B, C	43 (50.0)/43 (50.0)
α fetoprotein, ng/mL	137.0 [7.5–4814.0]
Advanced fibrosis, n (%) *	47 (54.7)
CTP class, A/B/C, n (%)	76 (88.4)/9 (10.4)/1 (1.2)
Biomarker profiles, expression level	
hsa_circ_0004018, normal tissue	0.0006 [0.0003–0.0009]
hsa_circ_0004018, tumor	0.0003 [0.0001–0.0006]
hsa_circ_0003570, normal tissue	0.0009 [0.0007–0.0013]
hsa_circ_0003570, tumor	0.0006 [0.0004–0.0012]
Body composition profile	
Skeletal muscle mass index, cm^2^/m^2^	48.8 [42.4–53.0]
Subcutaneous adipose tissue index, cm^2^/m^2^	38.6 [24.4–48.8]
Visceral adipose tissue index, cm^2^/m^2^	66.7 [50.2–86.1]
Sarcopenia, n (%)	48 (55.8)
Visceral adiposity, n (%)	27 (31.4)
Clinical outcomes	
Death, n (%)	50 (58.1)
Progression, n (%)	63 (73.3)
Duration of overall survival, mon	73.6 [12.3–133.1]
Duration of progression-free survival, mon	34.4 [9.8–85.5]

Values are expressed as median (interquartile range) or number (n, %). HCC, hepatocellular carcinoma; INR, international normalized ratio; BCLC, Barcelona Clinic Liver Cancer; CTP, Child–Turcotte–Pugh. * Patients with a Fibrosis-4 index ≥ 2.67 were defined as having advanced fibrosis.

**Table 2:** Clinical characteristics according to the expression levels of hsa_circ_0004018 and hsa_circ_0003570.

Variable	hsa_circ_0004018 (*n* = 86)			hsa_circ_0003570 (*n* = 86)		
	Low (*n* = 43)	High (*n* = 43)	*p*-Value	Low (*n* = 43)	High (*n* = 43)	*p*-Value
Age	56.0 [51.5–65.5]	58.0 [52.5–62.0]	0.662	54.0 [49.5–62.5]	58.0 [54.4–66.5]	0.07
Male, n (%)	36 (83.7)	37 (95.0)	>0.99	38 (88.4)	35 (81.4)	0.547
Body mass index, kg/m^2^	23.6 [21.8–25.8]	23.5 [21.6–25.1]	0.686	23.6 [21.8–25.0]	23.1 [21.5–26.0]	0.959
Liver function profiles						
Total bilirubin, mg/dL	0.6 [0.5–0.9]	0.8 [0.6–1.2]	0.158	0.8 [0.5–1.3]	0.7 [0.5–1.0]	0.749
Serum albumin, g/dL	3.8 [3.3–4.2]	3.9 [3.5–4.1]	0.606	3.8 [3.5–4.1]	3.9 [3.5–4.2]	0.208
Prothrombin time, INR	1.2 [1.1–1.2]	1.1 [1.0–1.2]	0.138	1.2 [1.1–1.2]	1.1 [1.0–1.2]	0.058
Fibrosis-4 index	3.0 [1.7–5.0]	3.2 [1.9–4.7]	0.85	3.2 [2.0–5.4]	2.4 [1.8–4.4]	0.27
Tumor profiles						
TNM stage, I/II/III/IV, n	24/4/10/5	17/7/12/7	0.47	18/3/14/8	23/8/8/4	0.119
BCLC, 0,A/B,C, n (%)	21 (48.8)/22 (51.2)	22 (51.2)/21(48.8)	>0.99	16 (37.2)/27 (62.8)	27 (62.8)/16 (37.2)	0.031
α-fetoprotein, ng/mL	127.3 [5.4–8050.0]	144.7 [10.8–3364.0]	0.933	1000.0 [35.4–16545.5]	22.1 [6.6–269.3]	0.009
Advanced fibrosis, n (%)	24 (55.8)	23 (53.5)	>0.99	28 (65.1)	19 (44.2)	0.083
CTP class, A/B,C, n (%)	39 (90.7)/4 (9.3)	37 (86.0)/6 (14.0)	0.737	37 (86.0)/6(14.0)	39 (90.7)/4 (9.3)	0.737
Body composition profiles						
Sarcopenia, n (%)	26 (60.5)	22 (51.2)	0.515	26 (60.5)	22 (51.2)	0.515
Visceral adiposity, n (%)	17 (39.5)	10 (23.3)	0.163	15 (34.9)	12 (27.9)	0.642

BCLC, Barcelona Clinic Liver Cancer; CTP, Child–Turcotte–Pugh.

**Table 3:** Prognostic factors for overall survival and progression-free survival according to each biomarker in patients with hepatitis B virus-related hepatocellular carcinoma.

Variable	Overall Survival				Progression-Free Survival			
	Univariate *p*-Value	Multivariate			Univariate *p*-Value	Multivariate		
		Hazard Ratio	95% Confidence Interval	*p*-Value		Hazard Ratio	95% Confidence Interval	*p*-Value
Age	0.138				0.047			
Male (yes/no)	0.309				0.255			
T stage (T3,4 vs. T0,1,2)	<0.001	6.142	3.081–12.242	<0.001	<0.001	5.040	2.547–9.974	<0.001
N stage (N1 vs. N0)	<0.001	3.397	1.172–9.849	0.024	<0.001			
M stage (M1 vs. M0)	<0.001	2.506	1.062–5.913	0.036	<0.001			
α-fetoprotein (≥200 ng/mL)	<0.001				<0.001			
CTP class (B, C vs. A)	<0.001	4.107	1.786–9.444	<0.001	<0.001	2.908	1.345–6.285	0.007
Advanced fibrosis	0.002				0.016			
Sarcopenia	0.114	2.026	1.103–3.722	0.023	0.164			
Visceral adiposity	0.128				0.029	0.445	0.238–0.830	0.011
hsa_circ_0004018 (high vs. low)	0.318				0.195	0.435	0.242–0.782	0.005
hsa_circ_0003570 (high vs. low)	0.005	0.437	0.235–0.813	0.009	0.039			

CTP, Child–Turcotte–Pugh.

**Table 4:** Clinical characteristics according to the combination and non-combination groups.

Variable	No Combination Groups (*n* = 55)	Combination Groups(*n* = 31)	*p*-Value
Age	57.0 [51.5–64.0]	58.0 [55.0–65.5]	0.134
Male, n (%)	48 (87.3)	25 (80.6)	0.610
Body mass index, kg/m^2^	23.7 [21.8–25.8]	23.1 [23.7–24.8]	0.284
Liver function profiles			
Total bilirubin, mg/dL	0.7 [0.5–1.2]	0.8 [0.6–1.1]	0.269
Serum albumin, g/dL	3.8 [3.3–4.2]	3.9 [3.6–4.2]	0.339
Prothrombin time, INR	1.1 [1.1–1.2]	1.1 [1.0–1.2]	0.083
Fibrosis-4 index	3.0 [1.7–5.0]	2.6 [1.9–4.7]	0.957
Tumor profiles			
TNM stage, I/II/III/IV, n	27/6/14/8	14/5/8/4	0.913
BCLC, 0,A/B,C	25 (45.5)/30 (54.5)	18 (58.1)/13 (41.9)	0.369
α-fetoprotein, ng/mL	223.4 [8.4–8359.0]	70.0 [7.5–365.9]	0.184
Advanced fibrosis, n (%)	32 (58.2)	15 (48.4)	0.515
CTP class, A/B,C, n (%)	48 (87.3)/7 (12.7)	28 (90.3)/3 (9.7)	0.942
Body composition profiles			
Sarcopenia, n (%)	31 (56.4)	17 (54.8)	1.000
Visceral adiposity, n (%)	19 (34.5)	8 (25.8)	0.551
Clinical outcomes			
Death, n (%)	35 (63.6)	15 (48.4)	0.251
Progression, n (%)	42 (76.4)	21 (67.7)	0.539
Duration of overall survival, mon	33.9 [9.3–130.8]	119.0 [45.1–133.9]	0.093
Duration of progression-free survival, mon	20.5 [8.04–58.6]	59.9 [19.8–124.2]	0.024

BCLC, Barcelona Clinic Liver Cancer; CTP, Child–Turcotte–Pugh.

**Table 5:** Prognostic factors for overall survival and progression-free survival according to the combined biomarkers in patients with hepatitis B virus-related hepatocellular carcinoma.

Variable	Overall Survival				Progression-Free Survival			
	Univariate *p*-Value	Multivariate			Univariate *p*-Value	Multivariate		
		Hazard Ratio	95% Confidence Interval	*p*-Value		Hazard Ratio	95% Confidence Interval	*p*-Value
Age	0.138				0.047			
Male (yes/no)	0.309				0.255			
T stage (T3,4 vs. T0,1,2)	<0.001	7.613	3.792–15.284	<0.001	<0.001	5.253	2.612–10.564	<0.001
N stage (N1 vs. N0)	<0.001	3.398	1.178–9.806	0.024	<0.001			
M stage (M1 vs. M0)	<0.001	2.645	1.120–6.249	0.027	<0.001			
α-fetoprotein (≥200 ng/mL)	<0.001				<0.001			
CTP class (B,C vs. A)	<0.001	3.827	1.722–8.504	0.001	<0.001	3.475	1.616–7.470	0.001
Advanced fibrosis	0.002				0.016			
Sarcopenia	0.114	2.034	1.110–3.728	0.022	0.164	1.895	1.094–3.282	0.023
Visceral adiposity	0.128				0.029	0.435	0.233–0.814	0.009
Combination group	0.082	0.399	0.209–0.765	0.005	0.195	0.422	0.237–0.751	0.003

CTP, Child–Turcotte–Pugh.

## Data Availability

The data used to support the findings of this study are available from the corresponding authors upon request.

## Supplementary Materials

The following supporting information can be downloaded at: https://www.mdpi.com/article/10.3390/genes14101963/s1, Supplementary Figure S1: Expression of GAPDH.
