# Supplementary figures and images for: Tissue Circular RNA_0004018 and 0003570 as Novel Prognostic Biomarkers for Hepatitis B-Related Hepatocellular Carcinoma
Source: Genes (Basel). 2023 Oct 20;14(10):1963. doi: 10.3390/genes14101963 (PMC10606672; doi:10.3390/genes14101963)

# GAPDH

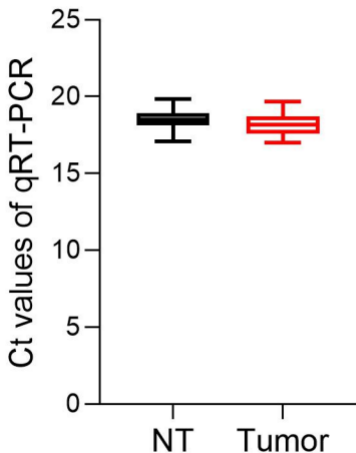

Supplement: Supplementary file 1 [file genes-14-01963-s001.zip › genes-2654914-supplementary.pdf]
